# A novel locus in *CSMD1* gene is associated with increased susceptibility to severe malaria in Malian children

**DOI:** 10.3389/fgene.2024.1390786

**Published:** 2024-05-24

**Authors:** Delesa Damena, Amadou Barry, Robert Morrison, Santara Gaoussou, Almahamoudou Mahamar, Oumar Attaher, Djibrilla Issiaka, Yahia Dicko, Alassane Dicko, Patrick Duffy, Michal Fried

**Affiliations:** ^1^ Molecular Pathogenesis and Biomarkers Section, Laboratory of Malaria Immunology and Vaccinology, National Institute of Allergy and Infectious Diseases, National Institutes of Health, Bethesda, MD, United States; ^2^ Malaria Research and Training Center, University of Sciences Techniques and Technologies of Bamako, Bamako, Mali; ^3^ Pathogenesis and Immunity Section, Laboratory of Malaria Immunology and Vaccinology, National Institute of Allergy and Infectious Diseases, National Institutes of Health, Bethesda, MD, United States

**Keywords:** candidate gene, snps, severe malaria, complement control, CSMD1

## Abstract

**Background:**

*Plasmodium falciparum* malaria is still a leading cause of child mortality in sub-Saharan Africa. The clinical manifestations of malaria range from asymptomatic infection to severe disease. The variation in clinical presentation is partly attributed to host genetic factors with estimated narrow-sense heritability of 23%. Here, we investigate the associations between candidate gene polymorphisms and the likelihood of severe malaria (SM) in a cohort of Malian children.

**Methods:**

Based on our previous genome-wide association studies (GWAS) analysis, candidate genes were selected for in-depth analysis using several criteria including gene-level GWAS scores, functional overlap with malaria pathogenesis, and evidence of association with protection or susceptibility to other infectious or inflammatory diseases. Single Nucleotide Polymorphisms (SNPs) residing within these genes were selected mainly based on *p*-values from previous severe malaria susceptibility GWAS studies and minor allele frequency (MAF) in West African populations.

**Results:**

Of 182 candidate genes reported in our previous study, 11 genes and 22 SNPs residing in these genes were selected. The selected SNPs were genotyped using KASP technology in 477 DNA samples (87 SM and 390 controls). Logistic regression analysis revealed that a common intron variant, rs13340578 in CUB and Sushi Multi Domain (CSMD1) gene, is associated with increased odds of SM in recessive mode of inheritance (MAF = 0.42, OR = 1.8, 95% CI = [1.78, 1.84], *p* = 0.029). The SNP is in linkage disequilibrium (LD) with multiple variants with regulatory features.

**Conclusion:**

Taken together, the current study showed that an intron variant rs13340578, residing in *CSMD1* gene, is associated with increased susceptibility to malaria. This finding suggests that modified regulation of complement may contribute to malaria disease severity. Further studies are needed to identify the causal variants and the underlying molecular mechanisms.

## Introduction

Malaria is still one of the most important infectious diseases, causing 249 million cases and 608,000 deaths in 2022. Nearly 95% of malaria cases and deaths occurred in sub-Saharan Africa; 76% of total deaths were reported in under-5 children (WHO, 2022; Venkatesan, 2024). In 2022, more than 50% of all deaths occurred in four African countries including Nigeria (31%), the Democratic Republic of the Congo (12%), Niger (6%), and Tanzania (4%) (Venkatesan, 2024). In countries such as Ghana, Kenya, and Malawi where the first malaria vaccine RTS,S/AS01 has been implemented, reductions in severe malaria cases and a 13% decrease in early childhood deaths have been reported (Venkatesan, 2024).

*P. falciparum* infection is associated with various clinical outcomes ranging from asymptomatic parasitaemia and uncomplicated malaria to severe malaria (SM) (Miller et al., 2002). The major complications of SM include cerebral malaria, severe anaemia, respiratory distress, pulmonary oedema and acute renal failure (Miller et al., 2002). In malaria-endemic areas, only a subset of cases progress to severe malaria and death (Mackinnon et al., 2005). The variations in malaria clinical outcomes are partly attributed to host genetic factors with estimated narrow-sense heritability of 20%–25% (Mackinnon et al., 2005; Damena and Chimusa, 2020).

Identifying genetic variations associated with clinical presentation during malaria infection may contribute to a better understanding of molecular mechanisms associated with host-pathogen interactions, which influence susceptibility to and protection against the disease (Achidi et al., 2008; Alamad et al., 2024). A classic example is the observation that African populations lacking Duffy blood group antigen were protected against *P. vivax* infection, because Duffy antigen is required for *P. vivax* to invade erythrocytes (Miller et al., 1976). This lack of Duffy antigen expression on erythrocyte surface is now known to be caused by a regulatory SNP in the Duffy blood group, within the chemokine receptor (DARC) gene that is near fixation in sub-Saharan Africa but absent in non-African populations (Hamblin and Di, 2000).

In continuing efforts to better understand *P. falciparum* pathogenesis*,* several genome-wide association studies (GWASs) have been conducted in diverse malaria-endemic populations, primarily by the MalariaGEN consortium (Timmann et al., 2012; Band et al., 2015; Ravenhall et al., 2018; MalariaGEN, 2019). GWASs have replicated the known protective loci including sickle cell (*HBB*) and *ABO* blood group, and identified new variants in *ATP2B4* and Glycophorin regions. However, the cumulative heritability attributable to these loci constitute about 10% (MalariaGEN, 2019), suggesting additional genetic variations that influence malaria disease severity remained to be discovered.

Although GWASs have elucidated the genetic basis of susceptibility or resistance to SM, the method suffers from limitations including weak performance in genetically diverse African populations, lack of translation of associated loci into suitable biological hypotheses, and the well-known problem of missing heritability (Visscher et al., 2017; Damena et al., 2019). To address some of these challenges, we recently applied various computational methods to SM GWAS summary statistics datasets (N = 17,000) (MalariaGEN, 2019) and predicted plausible candidate genes (N = 182) with their respective biological pathways (Damena et al., 2021). However, these genes were mainly prioritized by *in silico* functional analysis based on GWAS-summary statistics and were not supported by clinical observations in affected communities. Here, we investigate the association of human candidate gene polymorphisms with the likelihood of having a severe malaria episode in a cohort of Malian children that were followed from birth for up to 5 years.

## Methods

### Selections of genes and SNPs

In our previous study, we applied a statistical functional analytical method to the largest severe malaria susceptibility GWAS dataset to date and identified the well-known malaria susceptibility genes and several novel genes (N = 182) (Damena et al., 2021). We used this list to down-select genes (Supplementary Table S1) based on the following criteria: genes with top gene level-GWAS score (*p* < 10^–9^) (Damena et al., 2021); functions related to malaria pathogenesis including inflammation, anemia, cell adhesion and homeostasis, and those with reported associations with resistance or susceptibility to other infectious and inflammatory diseases.

We then selected representative SNPs residing within these genes based on *p*-values in previous GWASs (MalariaGEN, 2019) and minor allele frequency (MAF) in West African populations (Miller et al., 1976). We first extracted all SNPs 200 kb upstream and downstream of each gene selected from the GWAS summary statistics dataset, meta-analyzed across diverse populations in malaria endemic regions (MalariaGEN, 2019) using a custom Python script. We then selected SNPs with lowest GWAS *p*-value within each candidate gene. We computed MAF and pairwise linkage disequilibrium (LD) of these SNPs in 1000 Genome project database (Auton et al., 2015) using West African populations including Gambia, Nigeria and Sierra Leone. Eventually, we selected representative SNPs based on MAF and LD profile for each gene (Supplementary Table S1).

### Clinical definitions and sampling

Blood samples were obtained from children enrolled at birth in the Immuno-Epidemiology (IMEP) project in Ouélessébougou, Mali between September 2011 and May 2015 as previously described (Mahamar et al., 2017). The protocol and study procedures were approved by the Institutional Review Board of the National Institute of Allergy and Infectious Diseases at the National Institutes of Health (ClinicalTrials.gov ID NCT01168271), and the Ethics Committee of the Faculty of Medicine, Pharmacy and Dentistry at the University of Bamako, Mali (Mahamar et al., 2017). Cases were children who experienced at least one SM episode, defined according to WHO criteria (Trampuz et al., 2003), except for severe anemia in which we used the protocol definition of hemoglobin <6 gr/dL. Controls included children that experienced mild malaria but no SM during the follow up period. The median age of the participants at the last visit was 159 and 152 weeks for cases and controls, respectively. Venous blood samples and biological data including parasite density, haematology, and other characteristics were collected. DNA was extracted using Qiagen kit (Qiagen, Qiagen Str. 1, 40724 Hilden, Germany) following the manufacturer’s protocol.

### Genotyping, quality control (QC) and data analysis

The selected SNPs were genotyped using Kompetitive Allele Specific PCR (KASP) technique in LGC company (Teddington, Queen’s Rd, United Kingdom) (He et al., 2014). The genotype dataset was transformed to PLINK format (Purcell et al., 2007) using a custom Python script. Standard quality filtering including sample relatedness, Hardy-Weinberg equilibrium, heterozygosity, SNP missingness, and sample missingness were performed using PLINK1.9 software as described elsewhere (Marees et al., 2018). Briefly, SNPs with MAF <0.05, genotyping missingness >0.05, and those deviating from Hardy-Weinberg equilibrium (*p* < 0.01) were removed. Samples with missingness >0.05 were removed. Logistic regression was used to analyze quality passed dataset (Purcell et al., 2007). We tested for associations between polymorphisms and the odds for severe malaria using a range of different genetic models of inheritance including additive, dominant and recessive.

### Prioritization and annotation of putative regulatory SNPs

We applied regulatory SNP analysis tools including LDproxy (Machiela and Chanock, 2015) and Haploreg v4.1 (Ward and Kellis, 2012) to identify putative regulatory SNPs in LD with the significant SNP. The regulatory SNPs were selected based on a pre-calculated LD structure using the African populations in 1,000 Genome reference panel version 3 (Auton et al., 2015). SNPs in LD with the significant SNP (*r* ^2^ > 0.6) within a genome window size of 250 kb upstream and downstream of the significant SNP locus were selected. In addition, we performed neutrality test statistics including TajmaD (Tajima, 1989) and iHS (Voight et al., 2006) using VCFtools (Danecek et al., 2011) and rehh (Gautier and Vitalis, 2012), respectively on genomic regions encompassing *CSMD1* gene in African population of 1000 Genome project version 3 (Auton et al., 2015).

## Results

### Study population

A total of 477 participants from the IMEP were included: 87 children with SM defined as cases, and 390 children that experienced non-severe malaria infection defined as controls. Characteristics of the study population and study area have been previously described (Mahamar et al., 2017). To minimize confounding effects, age at last visit, hemoglobin type and ethnic group were matched for cases and controls (Table 1).

**TABLE 1:** Demographic data and haemoglobin type of study participants selected from Immuno-Epidemiology (IMEP) project in Ouélessébougou, Mali between September 2011 and May 2015 (Mahamar et al., 2017).

		Case (N)	Control (N)
Gender	Male	42	179
	Female	45	211
Ethnic group	Bamanan	77	320
	Malinke	4	15
	Fulani	4	17
	Soninke	2	7
	Others		31
Median age at last visit	Week	159	152
Haemoglobin type	AA	72	380
	AC	3	7
	AS	2	3

### Candidate gene and SNP selections

**FIGURE 1:**
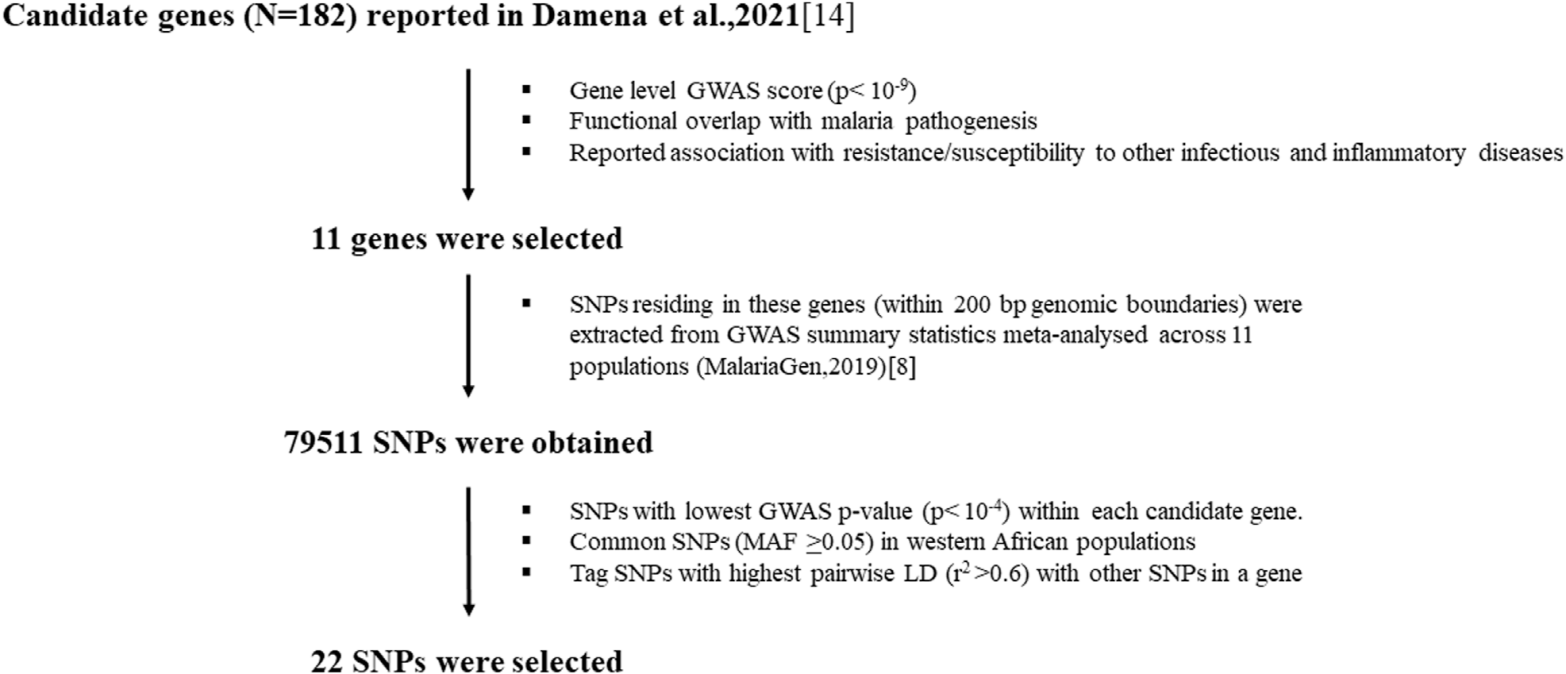
Workflow for the selection of candidate genes and SNPs.

Out of 182 candidate genes reported in our previous study (Damena et al., 2021), we down-selected eleven genes (Figure 1; Supplementary Table S1) based on criteria including GWAS scores, and evidence of association with protection or susceptibility to other infectious and inflammatory diseases. These genes included *CSMD1, CNTN4, FLT4, CAMK1D, NKAIN2, TSHD7B, CNTN5, KCNP, TMEM132, DLGAP1* and *DLGAP1*. Our previous *in silico* functional analysis showed that these genes are associated with different functions important in malaria disease such as regulation of inflammation (CSMD1), neural adhesions (*CNTN4,* *NKAIN2*, *CNTN5*, *TMEM132*, *DLGAP1*), vascular epithelial development (FLT4) and kinases (*CAMK1D, TSHD7B*) (Damena et al., 2021). We selected representative SNPs (N = 22) residing within these genes primarily based on *p*-values in previous GWASs (MalariaGEN, 2019) and MAF in west African populations (Supplementary Table S1).

### Quality control and association analysis

**FIGURE 2:**
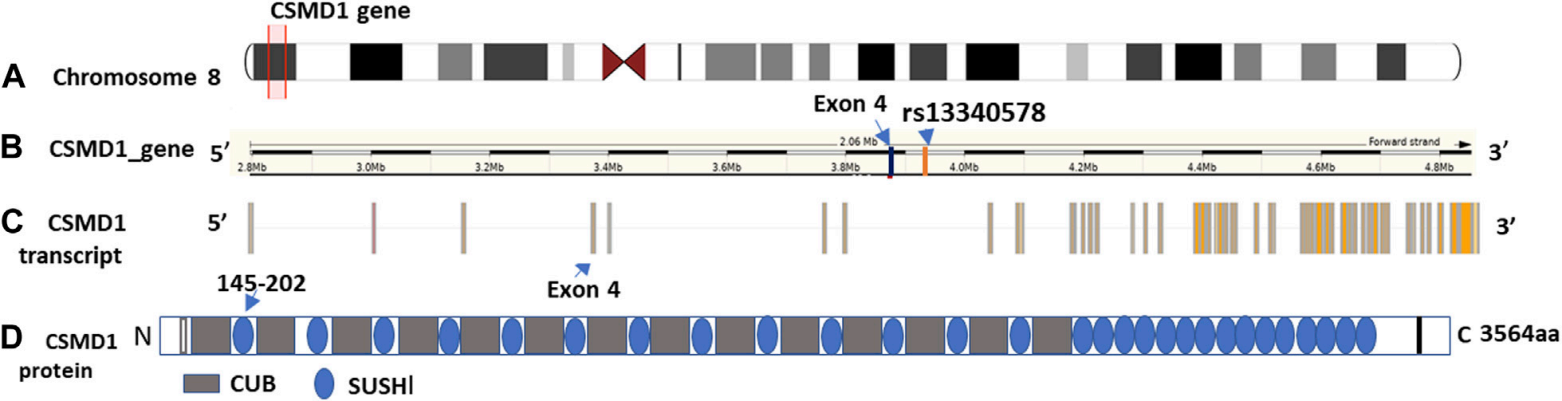
Genomic features and domain structure of CSMD1 gene. The genomic features are plotted using Gvis package in R software based on Ensembl GRCh37 annotations. **(A)** Location of CSMD1 gene on chromosome 8p13 highlighted in red (position: 2792875-4851494); **(B)** Location of rs13340578 on CSMD1 gene 63.5 kb upstream to exon 4: ENSE00001541898 (3889621-3889427); **(C)** CSMD1 transcript and **(D)** Domain structure of CSMD1 gene. CUB domain is indicated by grey rectangle and Sushi domain is indicted by blue circles. The sushi domain (145-292aa) encoded by Exon 4 (ENSE00001541898) is indicated by arrow.

Out of 477 total samples, 32 (27 controls and 5 cases) were removed due to missing genotype data (missing in greater than 5% genotypes). No SNPs were removed as a result of quality filtering. Upon alignment to the human reference genome (build 37- GRCh37), none of the variants were changed due to allele mismatch. After quality filtering, a total of 445 samples (362 controls and 83 SM) and 22 SNPs were retained for further analysis, with a total genotyping rate of 99.5%. Logistic regression analysis revealed that a common intron variant, rs13340578 in Sushi domain of *CSMD1* gene, is associated with increased odds of severe malaria in recessive mode of inheritance (MAF = 0.42, OR = 1.8, 95% CI = [1.78, 1.84], *p* = 0.029) (Table 2; Supplementary Table S2). The association remained significant after adjusting for multiple testing by permutation method (*p* = 0.04) (Table 2). The SNP is located 63.5 kb upstream to an exon (ENSE00001541898) that encodes Sushi domain of CSMD1 gene (Figure 2). The locus is approximately 800 Kb downstream of the previously reported SNPs (N = 5) that were associated with SM in Tanzanian populations (Ravenhall et al., 2018). rs13340578 is not in LD with any of these 5 SNPs described in Tanzania. We did not genotype these SNPs in the current study as they did not fulfil our selection criteria which are mainly based on the findings of GWAS meta-analysis across diverse populations (MalariaGEN, 2019).

**TABLE 2:** Logistic regression analysis of rs13340578 association with SM in a cohort of Malian children.

Chr	Position (GRCh37)	Major allele	Minor allele	MAF	MOI	OR	95% CI	*p*-value	Adj *p*-value (1000 permutation)
8	3952966	C	T	0.42	REC	1.81	[1.78,1.84]	0.029	0.04
					DOM	0.84	[0.83,0.86]	0.51	
					ADD	1.1	[1.11,1.14]	0.46	

MAF, minor allele frequency; MOI, mode of inheritance; OR, odds ratio; CI, confidence interval; REC, recessive; DOM, dominance; ADD, additive.

The *CSMD1* gene is located on the short arm of chromosome 8 (chr8:2,935,353-4,994,972) and is composed of 14 CUB and 15 Complement Control Proteins (Sushi) (Figure 2).

### Prioritization and annotation of putative regulatory SNPs in linkage disequilibrium with rs13340578

**FIGURE 3:**
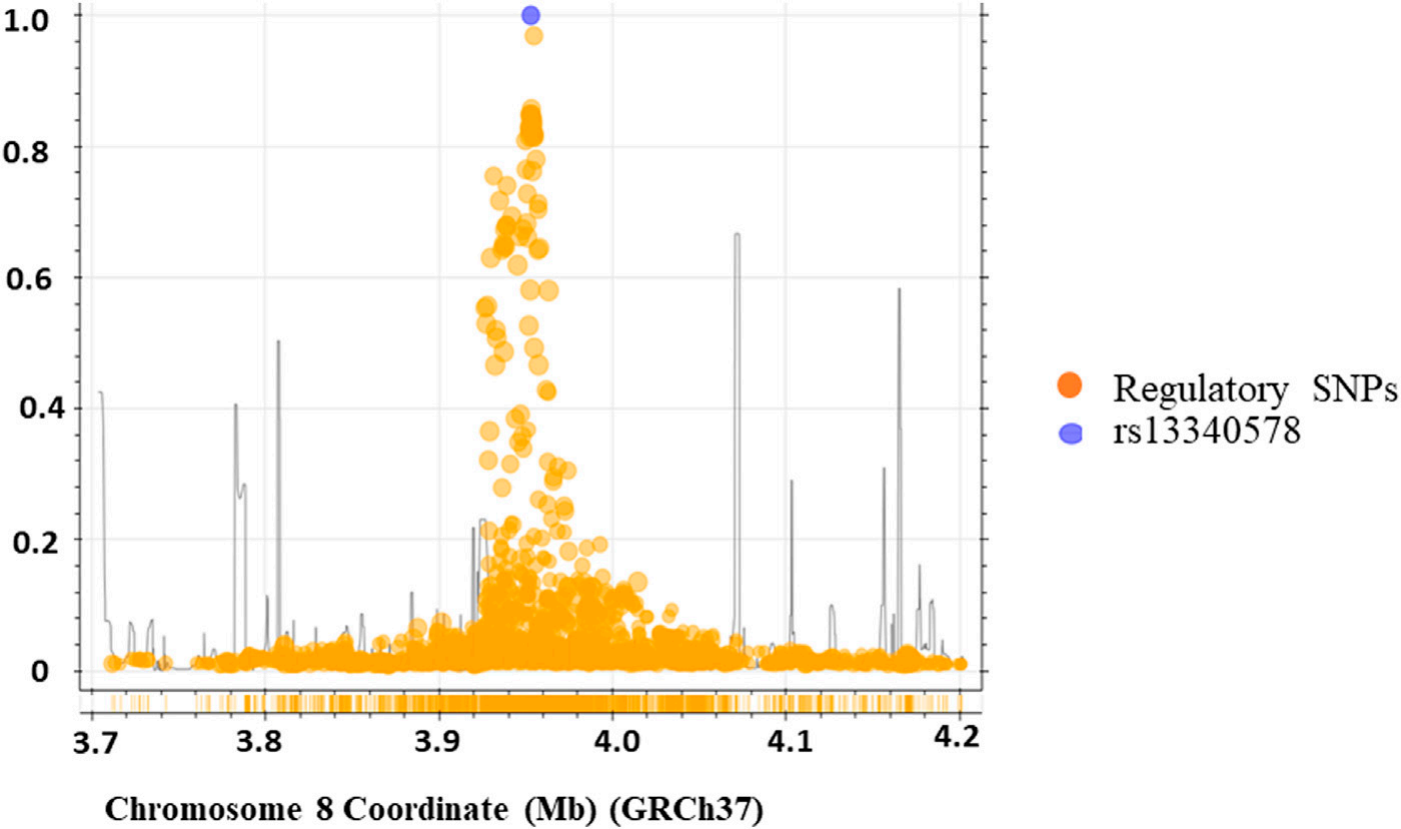
Genomic region containing the rs13340578 (250 kb upstream and downstream of rs13340578) identified by LD-proxy and Haploreg tools that explore proxy and putatively functional variants for a query variant based on a pre-calculated LD structure using the African reference population of 1,000 Genomes v.3. The purple circle indicates rs13340578 and the yellow circles indicate regulatory SNPs in the region.

We applied regulatory SNP analysis tools including LDproxy (Machiela and Chanock, 2015) and Haploreg v4.1 (Ward and Kellis, 2012) on African populations in 1000 Genome project to identify putative regulatory SNPs in LD with rs13340578 as described in Methods section. The Haploreg analysis identified 43 putative regulatory SNPs at *r* ^2^ > 0.6 in a genome window size of 250 kb upstream and downstream of rs13340578 (Supplementary Table S3). The LDproxy tool replicated all the SNPs identified by the Haploreg method plus additional 11 SNPs at the same threshold of *r* ^2^ > 0.6 (Figure 3; Supplementary Table S4). The two most frequent haplotypes consisted of that containing the major alleles (61.2%) and that containing the minor alleles (32.5%). Haplotype map of the regulatory variants in strong LD (*r* ^2^ > 8) with rs13340578 is shown in Supplementary Figure S1.

**FIGURE 4:**
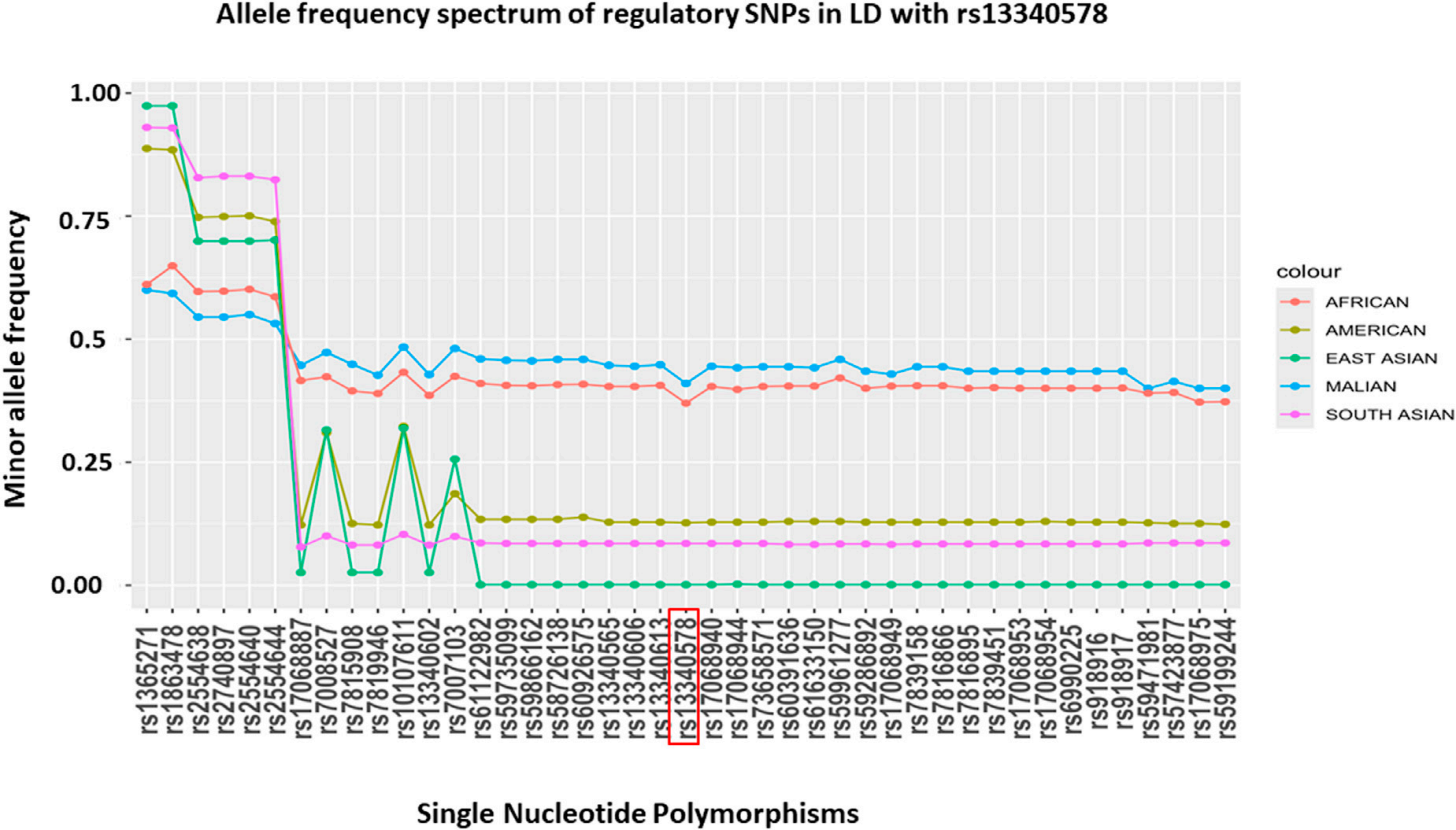
MAF of putative regulatory SNPs in LD (r2 > 6) with rs13340578 that were identified by LDproxy (Purcell et al., 2007) and Haploreg v4.1 (Marees et al., 2018) tools using African populations in 1000 Genome project v.3. The reference locus (rs13340578) is highlighted in red and the SNPs are ordered based on their genetic distance up-stream and downstream of rs13340578.

To assess the allele frequency spectrum of SNPs identified by the two methods (N = 54) across different populations, we obtained their MAF in Malian and global populations from GWAS dataset (MalariaGEN, 2019) and 1000 Genome project version 3, respectively (Auton et al., 2015) (Figure 4). We noted all of these SNPs are common in Malian (MAF = 0.4-0.6) and African continental (MAF = 0.37-0.65) populations. The majority (88.9%, N = 48/54) of SNPs had highest MAF in Malian populations followed by African continental populations, while they had lower frequencies in American and Asian continental populations (Figure 4). The MAF of these SNPs remains higher in the African population compared to Asian population, where SM is prevalent including India (Gujarati, Telugu), Pakistan (Punjabi), Vietnam (Minh) and Sri Lanka (Tamil) (Supplementary Figure S2). However, we did not detect signals of natural selection in the genomic region encompassing *CSMD1* gene in the African population of the 1000 Genome project version 3 (Supplementary Figure S3).

## Discussion

In this study, we systematically selected plausible novel candidate genes from our previous study (Damena et al., 2021) and performed candidate gene analysis in a cohort of Malian children. As African populations have high genetic diversity, severe malaria susceptibility variants have been shown to differ in allele frequencies, LD structure and effect sizes in sub-populations within endemic areas (Teo et al., 2010; Esoh et al., 2023). For instance, *HbS,* (rs334) is present at different frequencies in sub-Saharan Africa. It is prevalent at a frequency of ∼15% in northern Angola, Gabon and in southwestern Nigeria and east of Lake Victoria (Piel et al., 2013a). Frequencies of 7.5%–12.5% were predicted in large areas in West Africa extending from southern Senegal to northern Liberia, and from southern Ghana to northern Zambia. While *HbC* allele is largely absent in the South and Horn of Africa (Piel et al., 2013a), it is common in some parts of West Africa such as Mali, Burkina Faso, Ghana, Togo and Benin, though absent in other West African countries such as Cameroon and Chad, and East Africa (Piel et al., 2013b). This underscores the need for population-specific studies to capture severe malaria susceptibility variants that might be unique to an individual population.

In the current study, we identified a novel association between an intron SNP, rs13340578 in *CSMD1* gene and increased odds of severe malaria. The SNP is distant from the previously reported SNPs located in the same gene in Tanzanian populations (Ravenhall et al., 2018). Allelic heterogeneity in malaria-endemic regions has been well described for genes encoding red blood cell proteins such as β globin that influence susceptibility to SM (Bauduer, 2013) The genomic region containing rs13340578 is composed of multiple SNPs with regulatory features. This might suggest the association is driven by linked SNPs or group of SNPs which might alter the expression of the *CSMD1* gene. The MAF of these SNPs is generally higher in African populations, particularly in Mali. This may suggest the greater impact of these SNPs on health and susceptibility to diseases in the study population. The common-disease common-variant (CDCV) hypothesis depicts that common traits are most likely influenced by common variants with small to modest effects on diseases that may have escaped selection pressure (Risch and Merikangas, 1996). The differences in allele frequency in different populations is due to various demographic and evolutionary events in different parts of the world at different time points in history (Choudhury et al., 2014).

*CSMD1* is a multiple domain gene consisting of 71 exons and spans a 2 MB DNA region on the short arm of chromosome 8 (8p23.2) (Sun et al., 2001). The gene is composed of 14 N-terminal CUB domains that are separated from each other by a short consensus repeat (SCR) followed by 15 tandem SCR domains, a transmembrane domain, and a short cytoplasmic tail (Kraus et al., 2006). *CSMD1* is predominantly expressed in epithelial tissues and the central nervous system (CNS) (Uhlén et al., 1979). The gene is known to be an important regulator of complement activation and inflammation in the CNS (Kraus et al., 2006; Gialeli et al., 2018).

Complement is a system of plasma proteins that constitute a major component of the innate immune systems. Activation of complement leads to proteolytic cascades, which results in opsonization and lysis of the pathogen as well as in the generation of the classical inflammatory response through the production of potent proinflammatory molecules (Dunkelberger and Song, 2010; West et al., 2024). Complement can be activated by the classical, alternative, and mannose-binding lectin (MBL) pathways as described elsewhere (Rathnayake et al., 2021). All three complement pathways were reported to be activated during malaria infection by recognition of parasite and parasite-driven proteins in the host (Rathnayake et al., 2021). However, the malaria parasite has been known to escape host complement attack as a survival strategy (Rathnayake et al., 2021).

Apart from its protective role, activation of the complement pathway can also cause excess inflammation and extensive damage to self-tissues (Dunkelberger and Song, 2010). To prevent such damages, complement activation is tightly regulated by soluble and membrane-bound complement regulatory proteins (CRPs) at different points. *CSMD1* inhibits the classical and lectin pathways of complement by promoting enzymatic cleavage of the activated C4b and C3b. Fragments one and two of *CSMD1* bind C4 and C3 and facilitate their degradation by Factor I (Kraus et al., 2006). Thus, downregulation of this gene leads to elevated complement activation and associated pathology. Several studies have shown that variants and mutations in *CSMD1* genes are linked to different pathological conditions including susceptibility to neurodegenerative diseases, psychiatric disorders, infertility, and cancer (Liu et al., 2019). In schizophrenia sufferers, decreased *CSMD1* gene expression and its proteins predicted psychosis (Abd El Gayed et al., 2021) while increased complement (C4) expression predicted worse clinical outcomes after first psychosis (Mondelli et al., 2020).

The findings in the current study suggest that increased malaria disease severity might be due to dysregulation of complement. We propose the following model in which functional SNPs in the Sushi domain of *CSMD1* gene might downregulate gene expression in the brain, which in turn can lead to elevated complement levels. The resulting hypercomplementemia may mediate excess inflammatory reactions and formation of membrane attack complex. Previous studies implicated complement component, *C5a*, as a driver of cerebral malaria pathogenesis (Patel et al., 2008) and foetal growth restriction due to placental malaria (Conroy et al., 2013). Further studies are needed to validate this hypothesis.

SM is a complex disease with different clinical presentations including cerebral malaria, severe malarial anaemia, and others which may arise from distinct pathophysiological processes. Sub-phenotype analyses were not conducted in this study due to lack of adequate power. Detecting an association with a particular SM phenotype may highlight whether complement pathway is involved with a specific SM syndrome. Additionally, haplotype association analysis was not investigated for the identified putative regulatory SNPs in the significant locus. Furthermore, causal variants and the underlying molecular mechanisms have not been elucidated. In conclusion, *CSMD1* variants that modify regulation of complement may contribute to malaria disease severity. Further studies are needed to identify causal variants in this locus and the molecular mechanisms that mediate SM.

## Data Availability

The original contributions presented in the study are included in the article/Supplementary Material, further inquiries can be directed to the corresponding author.

## Supplementary material

SUPPLEMENTARY FIGURE S1 Haplotype blocks and pairwise linkage disequilibrium of putative regulatory SNPs (N = 27) in strong LD (*r* ^2^ > 8) with rs13340578. The values within boxes are pair wise SNP correlation (D′), bright red boxes without numbers indicate complete LD (D′ = 1).

SUPPLEMENTARY FIGURE S2 MAF of putative regulatory SNPs in LD (*r* ^2^ > 6) in African population and Asian populations where SM is prevalent (including Indian (Gujarati, Telugu), Pakistan (Punjabi),Vietnam (Minh) and Sri Lankan (Tamil)). The reference locus (rs13340578) is highlighted in red and the SNPs are ordered based on their genetic distance up-stream and downstream of rs13340578.

SUPPLEMENTARY FIGURE S3 Analysis of natural selection in genomic region encompassing *CSMD1* gene in African population of 1000 Genome project version 3. **(A)**Q-Q plot of the data after quality filtering; **(B)** Distribution of the data (expected vs. observed) after quality filtering **(D)** iHS scores. *X*-axis represents genomic position; *Y*-axis represent iHS score of the SNPs; The broken lines indicate threshold of values (iHS = ± 4) for deviation from neutrality. **(D)** Histogram of Tajima D values. There is no deviation from the expected D scores under neutral variations (<-2 or >2).

SUPPLEMENTARY TABLE S1 Profile of selected candidate genes and selected SNPs residing in these genes.

SUPPLEMENTARY TABLE S2 Logistic regression analysis of the associations between selected human candidate genes polymorphisms and SM in a cohort of Malian children.

SUPPLEMENTARY TABLE S3 SNPs with regulatory features in LD (r > 0.6) with rs13340578 identified by Haploreg v.4 tool using African populations in 1000 Genome project v.3.

SUPPLEMENTARY TABLE S4 SNPs with regulatory features in LD (r > 0.6) with rs13340578 identified by LDproxy tool using African populations in 1000 Genome project v.3.
