# Supplementary material for: A novel locus in CSMD1 gene is associated with increased susceptibility to severe malaria in Malian children
Source: Front Genet. 2024 May 24;15:1390786. doi: 10.3389/fgene.2024.1390786 (PMC11157005; doi:10.3389/fgene.2024.1390786)
Supplement: Supplementary file 4 [file Table1.DOCX]

**Supplementary Table 1. Profile of selected candidate genes and selected SNPs residing in these genes**

| **Selected candidate genes** | | | | | **Selected SNPs** | | | | | | |
| --- | --- | --- | --- | --- | --- | --- | --- | --- | --- | --- | --- |
| **Gene** | **Chr** | **Start** | **End** | **Gene level P_value (Damena et al.,2021)** | **RS_ID** | **Genomic position** | **REF_Allele** | **ALT_Allele** | **P_vale_SNP (MalariaGen ,2019)** | **MAF** | **Biotype** |
| CSMD1 | 8 | 2792674 | 4852528 | 1.58E-12 | rs17136827 | 4014579 | C | G | 0.000011 | 0.19 | protein_coding |
|  |  |  |  |  | rs7000720 | 4007069 | T | C | 6.65E-05 | 0.45 | protein_coding |
|  |  |  |  |  | rs13340578 | 3952966 | C | T | 0.00010699 | 0.30 | protein_coding |
| CNTN4 | 3 | 2140349 | 3099845 | 3.88E-09 | rs113562487 | 2700902 | CT | C | 0.0014393 | 0.08 | protein_coding |
| FLT4 | 5 | 180028305 | 180076824 | 9.96E-08 | rs392227 | 180046934 | G | T | 2.37E-06 | 0.39 | CTCF_binding_site |
|  |  |  |  |  | rs448012 | 180046344 | G | C | 0.0000506 | 0.42 | CTCF_binding_site |
|  |  |  |  |  | rs382266 | 180047412 | T | C | 0.00013521 | 0.708 | protein_coding |
|  |  |  |  |  | rs366388 | 180041008 | T | C | 0.00052955 | 0.67 | protein_coding |
| CAMK1D | 1 | 12391382 | 12871933 | 1.87E-06 | rs17136827 | 12464973 | T | C | 0.0003411 | 0.195 | protein_coding |
|  |  |  |  |  | rs1644391 | 12868304 | A | C | 0.0041739 | 0.25 | protein_coding |
| NKAIN2 | 6 | 124124868 | 125146986 | 6.74E-09 | rs9491199 | 124923968 | A | G | 4.50E-06 | 0.08 | protein_coding |
| THSD7B | 2 | 137522914 | 138435487 | 7.84E-07 | rs56260128 | 138167165 | T | C(*AT) | 0.0030757 | 0.23 | protein_coding |
|  |  |  |  |  | rs13429534 | 137677982 | G | C | 0.0043275 | 0.09 | non-coding_transcript_variant |
|  |  |  |  |  | rs10166002 | 137857371 | T | C | 0.0062817 | 0.72 | protein_coding |
| CNTN5 | 1 | 98891505 | 100229816 | 1.28E-06 | rs4754664 | 99913789 | C | T | 0.00025714 | 0.77 | non_coding_transcript_variant |
|  |  |  |  |  | rs10750480 | 99939338 | T | C | 0.0004841 | 0.83 | non_coding_transcript_variant |
|  |  |  |  |  | rs7945782 | 99925256 | G | A | 0.00049487 | 0.76 | non_coding_transcript_variant |
| KCNIP1 | 5 | 169780680 | 170163836 | 2.64E-06 | rs6861762 | 169993388 | C | T | 0.002207 | 0.27 | protein_coding |
|  |  |  |  |  | rs59266801 | 169990427 | A | G | 0.0027597 | 0.27 | promoter_flanking_region |
|  |  |  |  |  | rs4867613 | 169994628 | C | T | 0.0069198 | 0.23 | protein_coding |
| TMEM132 | 12 | 128750947 | 129193460 | 2.18E-08 | rs1514902 | 128787775 | G | A | 0.0030829 | 0.37 | protein_coding |
| DLGAP1 | 18 | 3495829 | 4455466 | 2.38E-10 | rs510668 | 3521041 | T | C | 0.001392 | 0.73 | enhancer |
| EPHA7 | 6 | 94452021 | 9463561 | N/A | rs62418762 | 93218698 | C | T | 0.000006 | 0.06 | Intron variant |
