# Supplementary figures and images for: A novel locus in CSMD1 gene is associated with increased susceptibility to severe malaria in Malian children
Source: Front Genet. 2024 May 24;15:1390786. doi: 10.3389/fgene.2024.1390786 (PMC11157005; doi:10.3389/fgene.2024.1390786)

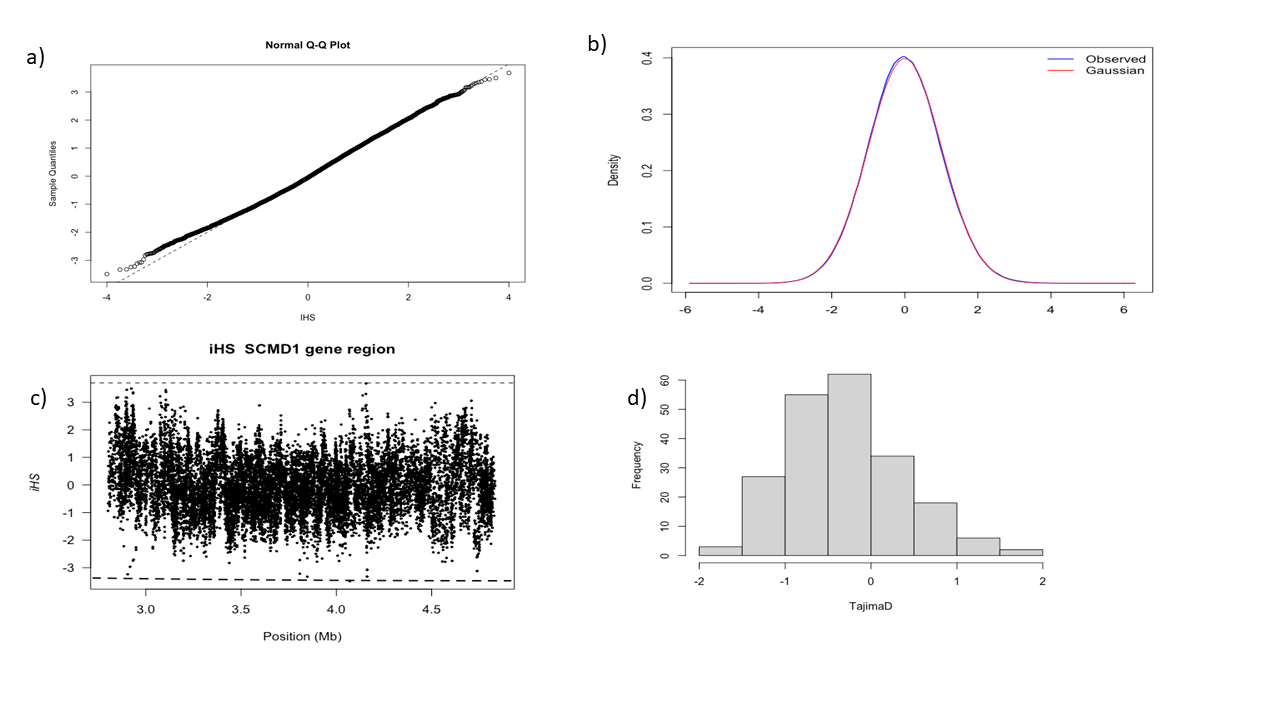

Supplement: Supplementary file 5 [file Image3.TIF]

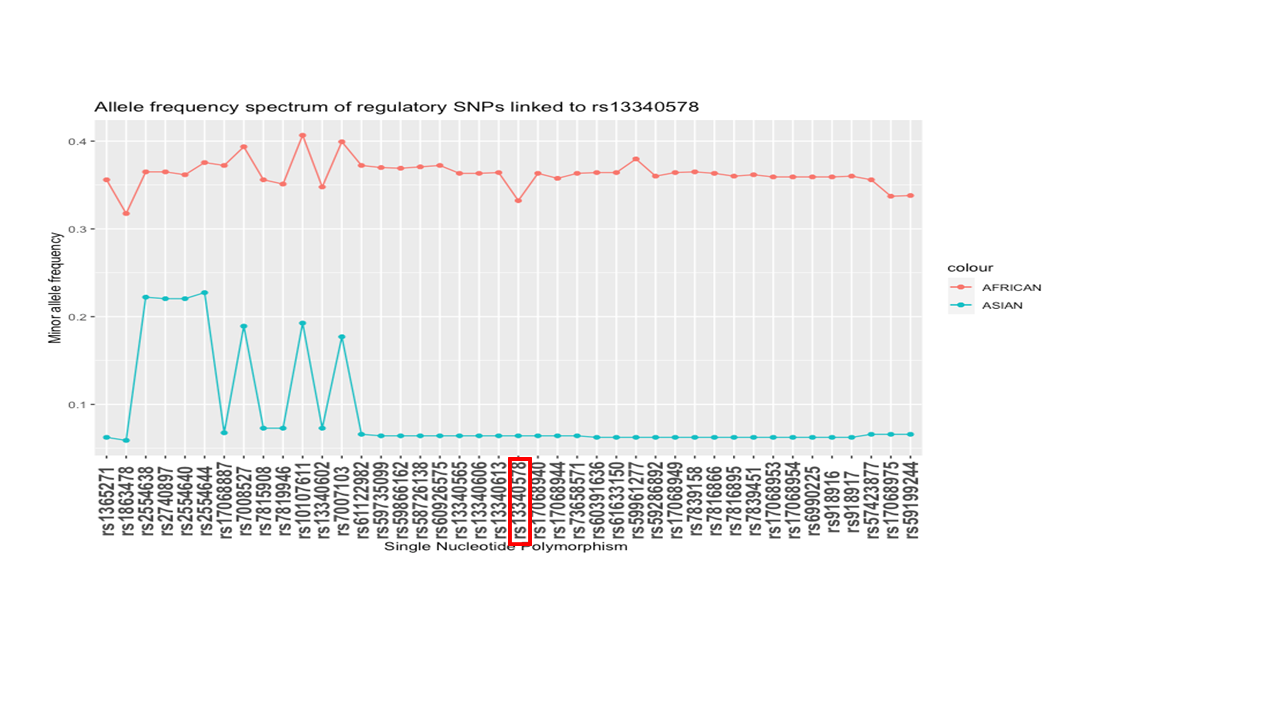

Supplement: Supplementary file 6 [file Image2.TIF]

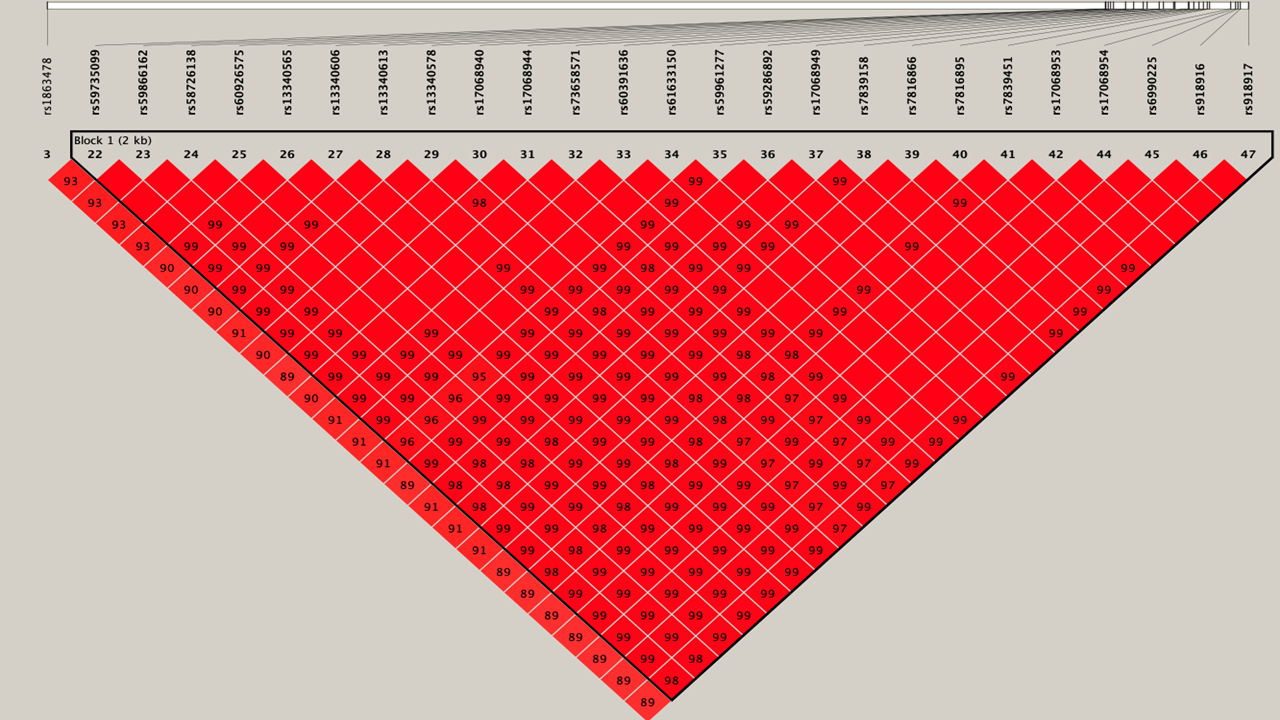

Supplement: Supplementary file 7 [file Image1.TIF]
